# Transparency and diversity to advance legal-scientific communication

**DOI:** 10.1016/j.fsisyn.2024.100557

**Published:** 2024-09-21

**Authors:** Jason M. Chin, Justice Belinda Baker

**Affiliations:** College of Law, Australian National University, Australia; ACT Supreme Court, Australia

We are honoured to provide this opening editorial for the special issue on ‘Rethinking Scientific Communication in Courts’.^1^ The four articles within this special issue contribute to ongoing international discussions and innovations about improving the ways in which scientific knowledge is conveyed for legal users [[1], [2], [3]].^2^ They arose from a conference of the same name held at the Australian National University in November 2023.^3^

The importance of clear and epistemically modest scientific communication for the administration of justice, particularly the administration of criminal justice, cannot be understated. As Michael Kirby AC CMG has observed, ‘human justice is always prone to error and mistaken outcomes’ [4]. Whilst the possibility of error or mistake is not limited to cases involving expert evidence, expert evidence is an area where courts are particularly vulnerable to error. The hard lesson of mounting Royal and Special Commissions is that the risks of wrongful convictions are real where the limitations of expert evidence are not appreciated, either by the courts, or by the experts themselves.

Accordingly, the conference was convened in recognition of the particular challenges that modern courts face when dealing with expert evidence. There has been an exponential growth in scientific research in many forensic areas. As the conference organisers recognised, much of this research is of uneven quality and there is often a lack of transparency in the research, which is necessary for the accurate assessment of its reliability.

There are two themes which emerged in the conference and which feature in the four articles. The first is the strength of disciplinary diversity, in particular, the importance of drawing upon the expertise of a range of experts in different disciplines in creating, compiling and communicating expert evidence. As the philosopher of science Naomi Orsekes has observed, this kind of intellectual diversity strengthens knowledge generation processes, including by allowing for the interrogation of assumptions that may otherwise slip through unnoticed and produce error. In particular, she notes: ‘In diversity there is epistemic strength [5].’ In this spirit, the special issue's authors come from a variety of fields, including forensic science, law, science policy and education, psychology, metaresearch, systematic review methods, and beyond [[6], [7], [8], [9]]. The authors rely on that diverse expertise to provide new ideas and solutions for the challenges of communicating science in legal contexts.

Second, the articles in this special issue highlight the importance of transparency in both producing and conveying scientific knowledge for the legal system. As psychologist and meta-scientist Simine Vazire has noted, users of scientific research need transparency so that they can distinguish between scientific claims that are safe to rely on and those that are not:

Without high levels of transparency in scientific publications, journal editors, reviewers, and readers of scientific manuscripts are in a similar position as buyers of used cars – they cannot reliably tell the difference between lemons and high quality findings [10, see also 11].

Similarly, many of the articles in this issue contend that the advancement of justice requires that scientific research adopt a high level of transparency. This includes an outline of Victoria Police's recent innovations in more transparently conducting its forensic work [7] and reflections on the broader relationship between transparency and trust [8].

## 1 Martire et al.: ‘Understanding ‘error’ in the forensic sciences: A primer’

The special issue begins with a large academic-practitioner collaboration led by Kristy A Martire [6]. It represents a joint venture of the Victoria Police Forensic Services Department and the Evidence-based Forensics Initiative, the latter being an interdisciplinary group of university researchers and scientific practitioners. The author list includes practicing crime scene examiners, toxicologists, fingerprint examiners, as well as many other practitioners and university researchers. The group applies that diverse expertise to decades of forensic scientific research studying the concept of ‘error’ to extract seven key lessons.

Martire and colleagues' methodology is worth highlighting. In short, Martire's team convened ten webinars in which university researchers were paired with forensic practitioners to present about an influential forensic science article before leading a discussion about that paper. They found that the multiplicity of perspectives produced valuable insights on the papers they discussed:

These presentations and the discussions that followed revealed diverging perspectives, provided methodological insights, suggested alternative interpretations, foregrounded implicit and sometimes faulty assumptions, and ultimately assisted us to develop a shared understanding of error in the forensic sciences [6].

From those presentations and discussions, the authors discerned the following seven key lessons about error:

1. There are different perspectives on what constitutes an error, therefore Error is subjective
2. There are different ways to compute or estimate the same error, therefore Error is multidimensional
3. All complex systems involve error, therefore Error is unavoidable
4. Some approaches to error management are more effective than others, therefore Error is cultural
5. Performance can be improved by attending to error, therefore Error is educational
6. Successful communication of error is challenging, therefore Error is misunderstood
7. Error management goes beyond the boundaries of any individual discipline, therefore Error is transdisciplinary [6].

As the authors conclude, these lessons, and the research underlying them, lay a foundation for future collaborative initiatives which may further shine a light on the concept of error in forensic science [6].

## 2 Ballantyne et al.: ‘A transparent approach: Openness in forensic science reporting’

Transparently acknowledging the possibility of error is also a major part of the second article, led by Kaye Ballantyne [7], which reports on Victoria Police Forensic Services Department's ‘annexure’ program. In short, the program has developed standard form attachments for each of the 52 services it provides (e.g., crime scene, fingerprints). These annexures include vital information, such as a review of the research supporting the practice (or a warning that limited research exists). They are appended to the reports that the forensic services department produces for lawyers and other users.

Ballantyne and colleagues' contribution to this issue goes beyond describing the annexure program to providing valuable transparency about its genesis and impacts. The authors note that Victoria Police began producing the information that would ultimately wind up in its annexures much earlier. This was done to comply with the Practice Note for expert evidence which came into effect in the Supreme and County Courts of Victoria in 2014. However, the Practice Note was intended to be ‘triggered on request’; a party was required to request a full expert report containing the required information. Surprisingly, requests for disclosure of this information came in only ‘a handful of cases over the 10 years since introduction – despite regular promotion of the value of the Practice Note and the type of material that would be provided to legal stakeholders including both prosecution and defence’ [7]. As a result, Ballantyne's group began including these annexures proactively – annexures that, again, transparently report the limits of their practices in many cases.

Ballantyne and colleagues conclude with a report of a small survey conducted amongst the forensic practitioners whose casework has been affected by the annexures. The respondents mostly viewed the annexures positively, with just over half reporting they were ‘helping them to better explain their science to a lay audience’ [7]. There is also some indication that the annexures are promoting more critical scrutiny of forensic evidence, with about a quarter of respondents saying they were now receiving more questions about their evidence in court [7].

As the authors observe, one difficulty going forward, will be in finding the resources to update the 52 annexures as ‘additional knowledge is gained through research and publications, and methods are continuously improved and updated’ [7]. It is important this updating occurs given the view of the clear benefits of the annexure program.

## 3 Chin et al.: ‘A plan for systematic reviews for high-need areas in forensic science’

Ballantyne and colleagues’ work forms a dyad with the next article, which calls for interdisciplinary teams to perform systematic reviews of high leverage forensic practices [9]. Indeed, as the authors (Chin and colleagues) note, when major decisions need to be made about medicine and education, those fields often turn to systematic reviews [12]. A key producer of systematic reviews for healthcare decision making is the 10.13039/501100024530Cochrane Collaboration, whose reviews provide important knowledge about the level of research-based support for drugs and other interventions. Surprisingly, as the authors lay out, there are no rigorous and transparent systematic reviews for courts to rely on in forensic science [9].

Chin and colleagues go on to discuss the problems that flow from forensic science's lack of high quality systematic reviews. For instance, the informal literature reviews produced by expert witnesses – as Ballantyne et al. note – have historically been incomplete and biased towards demonstrating the forensic practice is error free. This is precisely why annexures such as those produced by Victorian Police are needed. However, as mentioned above, they are difficult to keep up-to-date.

The authors then further detail the benefits of systematic reviews, such as those produced by Cochrane. Specifically, the authors describe how systematic reviews ‘use formal, explicitly stated methods to collate and synthesise findings of studies that address a clearly formulated question’ [9]. This will include detailing how the literature was searched, in which databases, and ideally presenting the full Boolean search strings used. As a result, the review is verifiable, reproduceable, and readily updateable.

The authors suggest that previous attempts to produce research reviews in forensic science have been hindered by a lack of disciplinary diversity and, in particular, a lack of research synthesis expertise (that is, experts trained by groups like Cochrane to produce systematic reviews). They call for forensic scientists to collaborate with both systematic review experts and specialists in scientific communication, with the latter providing knowledge about how to clearly draft and summarise systematic reviews so that they can be easily understood both by legal professionals and by lay people.

They conclude by observing that learned academics are well placed to organise these reviews because they are not aligned with any particular interest in the justice system and have access to leading scientists [9].

## 4 Heavey and Houck: ‘Rethinking scientific communication in courts: A question of credibility’

This special issue ends with Anna Heavey and Max Houck's big picture reflections on scientific communication in forensic science [8]. Through the leitmotif of ‘credibility’, their commentary reinforces many of the points made by other authors in this issue:

All of this emphasizes the need to ensure that the evidence is communicated and understood in a way that the court can make objective and measured sense of its relevance and weight to the matter at hand. Where this communication fails, or is later found lacking, it is often the credibility of the forensic science (and its communicator) that comes under scrutiny [8].

Heavey and Houck endorse ‘building robust and transparent systems’ within forensic science. As noted above, such transparency would include the disclosure of a field's limitations as promoted by Victoria Police's annexures [7], which could be founded by systematic reviews [9]. They also endorse investment in error detection because:

Forensic science is a high-risk, high-consequence field which brings with it an extremely low tolerance for error or learning by experimentation [8].

In this way, the they reinforce Martire and colleagues’ seven lessons towards better understanding and mitigating error [6].

Finally, the authors recommend greater collaboration within forensic science and between forensic science and other fields [8, citing 13]. We agree. Open dialogue embracing a diversity of views and backgrounds has proven essential towards advancing science. Its role in improving scientific communication in courts is no different.

## Funding disclosure

None.

## Conflict of interest

Chin: Chin is the registered reports editor of *Forensic Science International: Synergy* and editor of this special issue. This opening editorial has been handled by an independent editor.

## Footnotes

1 Chin convened and participated in the conference and special issue. Baker was not involved in the conference, but rather is commenting from the perspective of a judicial officer and former barrister.

2 https://www.sciencedirect.com/special-issue/102DNQD8KWT.

3 We thank the attendees and speakers of that conference. The speakers were: Rachael L. Brown, Simine Vazire, Gary Edmond, Matthew J. Page, Kristy A. Martire, Kaye Ballantyne, Anna Heavey, Max Houck, Mehera San Roque, and Anna-Maria Arabia.
